# A Voxel-Based Morphometry Study of the Brain of University Students Majoring in Music and Nonmusic Disciplines

**DOI:** 10.1155/2015/274919

**Published:** 2015-09-30

**Authors:** Kanako Sato, Eiji Kirino, Shoji Tanaka

**Affiliations:** ^1^Department of Information and Communication Sciences, Sophia University, Tokyo 102-8554, Japan; ^2^Department of Psychiatry, Juntendo University School of Medicine, Tokyo 113-8421, Japan; ^3^Juntendo Shizuoka Hospital, Shizuoka 410-2295, Japan

## Abstract

The brain changes flexibly due to various experiences during the developmental stages of life. Previous voxel-based morphometry (VBM) studies have shown volumetric differences between musicians and nonmusicians in several brain regions including the superior temporal gyrus, sensorimotor areas, and superior parietal cortex. However, the reported brain regions depend on the study and are not necessarily consistent. By VBM, we investigated the effect of musical training on the brain structure by comparing university students majoring in music with those majoring in nonmusic disciplines. All participants were right-handed healthy Japanese females. We divided the nonmusic students into two groups and therefore examined three groups: music expert (ME), music hobby (MH), and nonmusic (NM) group. VBM showed that the ME group had the largest gray matter volumes in the right inferior frontal gyrus (IFG; BA 44), left middle occipital gyrus (BA 18), and bilateral lingual gyrus. These differences are considered to be caused by neuroplasticity during long and continuous musical training periods because the MH group showed intermediate volumes in these regions.

## 1. Introduction

The brain is not a static organ, but it changes dynamically throughout an individual's lifespan. During development, the brain changes flexibly with various kinds of experiences. These changes are characterized both structurally and functionally [1, 2]. Structural changes include alterations in the volume of specific gray matter (GM) regions, as well as volume and fiber arrangement in the white matter (WM). Functional changes include alterations in regional activation and functional connectivity between regions. The musician's brain is regarded as an ideal model of brain plasticity, because musicians usually start musical training very early in life and continue for many years into adulthood [1, 2]. Musical training requires complex information processing or functions and includes auditory and somatosensory processing, motor control, attention, working memory, executive functions, and higher-order integrative functions. Playing musical instruments requires multitasking, in which emotional expression is critical. Musical training is also an activity in which procedural, episodic, and semantic memories and emotion converge. Therefore, many brain regions in distinct domains can be changed with musical training.

Magnetic resonance imaging (MRI) assesses anatomical structures in the GM and WM. Voxel-based morphometry (VBM) uses a voxel-wise analysis method for determining focal differences in volume [3]. Previous VBM studies have found GM volume differences between musicians and nonmusicians. A whole brain VBM study showed that professional musicians (keyboard players) have larger bilateral inferior temporal gyri (ITG), left Heschl's gyrus (HG.L), bilateral precentral gyri, right superior parietal cortex, left inferior frontal gyrus (IFG.L), right medial frontal gyrus, and left anterior cerebellar lobe than amateur musicians and nonmusicians [4]. Increased GM volumes in the HG.R, bilateral superior temporal gyri (STG), left middle temporal gyrus (MTG.L), ITG.R, right posterior cingulate gyrus (PCC.R), right central sulcus, right superior frontal gyrus (SFG.R), and IFG.R in musicians compared with those in nonmusicians were also observed in another study [5]. Other studies have reported a larger GM volume of the left IFG pars opercularis (BA 44) in male orchestra musicians compared with nonmusicians, which positively correlated with years of musical performance [6, 7]. In addition, the left hippocampus, right supplementary motor area (SMA.R), SFG.R, right middle frontal gyrus (MFG.R), PCC.L, right insula, and STG.L were positively correlated with the degree of musical practice [8]. Moreover, another VBM study [9] detected increased GM volumes with increased musical training intensity in the right fusiform gyrus (FG.R), right mid orbital gyrus, IFG.L, left inferior parietal lobule (IPL.L) (*P* < 0.001, uncorrected), and bilateral cerebellum Crus II. An increase in HG.L was also detected, but only at *P* < 0.005 (uncorrected). In contrast, decreased GM volumes were observed in bilateral perirolandic and striatal areas that are related to sensorimotor functions [9]. Thus, the brain regions that differ between musicians and nonmusicians depend on the study and have not been replicated well.

Because of the abovementioned variability in VBM results, corroborative analysis with different sets of participants is needed. Here, we report the results of our VBM analysis of whole brain structural data from university students majoring in music and those majoring in nonmusic disciplines. We chose university students because they are all in the same generation with regard to the cultural environment and exhibit less background variability than professional musicians of a wide range of ages. Students majoring in music studied in the same university as those majoring in other nonmusic disciplines. Our aim was to investigate how education in different disciplines (music versus nonmusic) affects brain structure. Because nonmusic major students still had various degrees of extracurricular music activities, we divided these students into two groups; therefore, we examined three groups in total: music expert (ME) group (music major students), music hobby (MH) group (nonmusic major students having active extracurricular music lessons), and nonmusic (NM) group (nonmusic major students with no or less musical training). Differences between the ME group and NM group suggest a direct effect of musical training. The MH group was included to provide insight into the effect of different degrees of musical training on brain structure.

## 2. Methods

### 2.1. Participants

This study was approved by the local ethics committees, the Sophia University Ethics Committee, and the Juntendo University Ethics Committee. All participants provided written informed consent before the study commenced. University students majoring in music (*n* = 23) and nonmusic disciplines (*n* = 32) were recruited. All participants were healthy right-handed Japanese females and had no history of neurological disorders. Students majoring in music started musical training at around 3–5 years of age, which continued to the present. They were screened on enrollment in a college of music. All of them specialized in classical music and played various instruments (piano, violin, cello, clarinet, or trumpet). Students majoring in nonmusic disciplines showed various degrees of extracurricular music activities and were therefore divided into two groups: students with active extracurricular music lessons and those with less musical training. Students in the former group had taken music lessons for nine years or longer, whereas those in the latter group had taken lessons for seven years or shorter. Thus, there were three groups of students: ME: music expert group (*n* = 23; age: 18–26 years; mean = 21.2 years), MH: music hobby group (*n* = 17; age: 18–23 years; mean = 20.9 years), and NM: nonmusic group (*n* = 15; age: 19–23 years; mean = 21.6 years).

### 2.2. Data Acquisition

Structural MRI was performed using a 3T Philips Achieva scanner at Juntendo Hospital, Tokyo, with a MPRAGE sequence (TE = 3.3 ms, TR = 15 ms, TI = 955.5 ms, and flip angle = 15°). MRI included a 3D set of T1 images (voxel size = 1 × 1 × 1 mm; FOV = 180 × 232 × 256 mm). Imaging time was 3 min 31 s. The scan protocol was identical for all participants.

### 2.3. Analysis

T1 images were preprocessed by VBM using SPM12 (Wellcome Department of Imaging Neuroscience, London, United Kingdom), which segments T1 brain images into GM, WM, and cerebrospinal fluid. GM images were then subjected to spatial normalization to standard MNI space using the DARTEL toolbox (Wellcome Department of Imaging Neuroscience) [10]. Individual voxel volumes were modulated by whole brain volume with proportional scaling. All images were smoothed using the Gaussian kernel of 8 × 8 × 8 mm FWHM. VBM analyzed differences in local GM and WM volumes across the whole brain using two-sample* t*-tests between ME and NM groups, and analysis of variance (ANOVA) across all three groups.

## 3. Results

**Figure 1:**
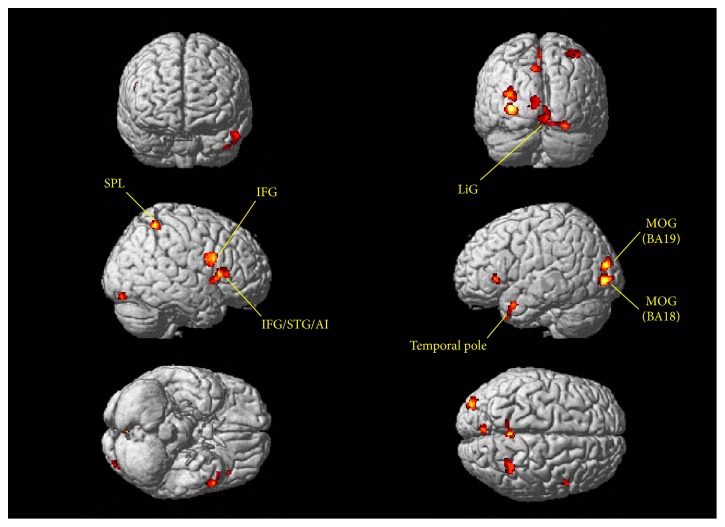
Brain regions with larger gray matter volumes in the ME group than in the NM group (*P* < 0.01, uncorrected). Only cluster sizes >100 voxels are shown. No surface regions were smaller in the ME group than in the NM group.

**Figure 2:**
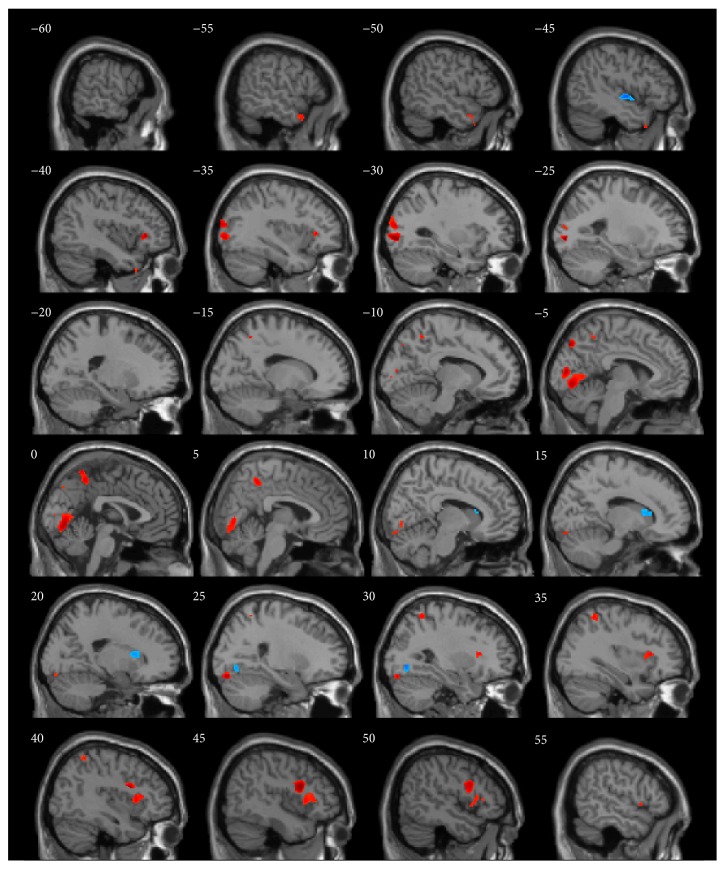
Sagittal-plane locations of brain regions with gray matter volumetric differences between ME and NM groups. Only clusters sizes >100 voxels are shown. Regions in red or blue color were larger or smaller, respectively, in the ME group.

**Figure 3:**
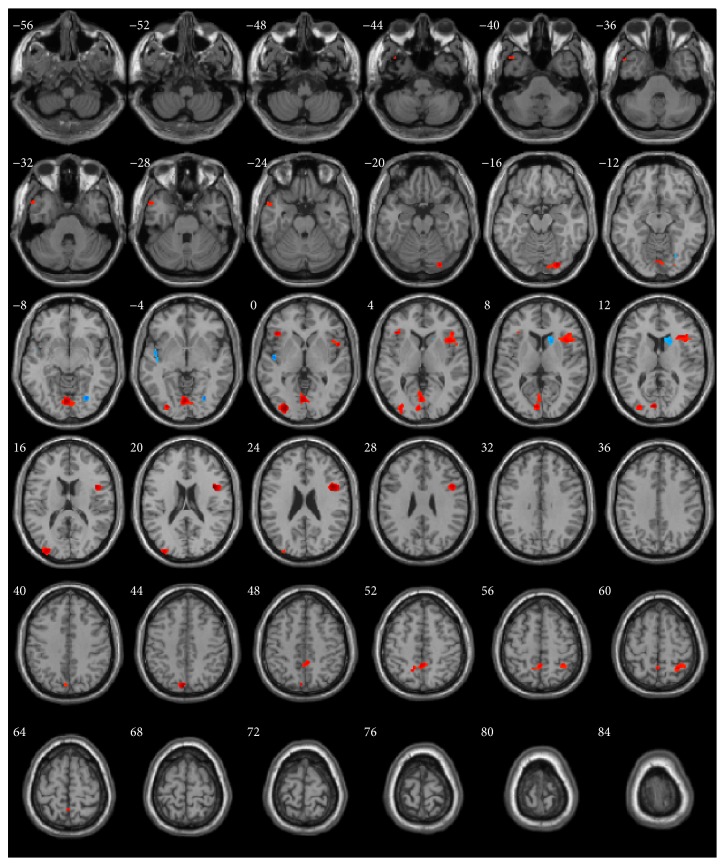
Horizontal-plane locations of brain regions with gray matter volumetric differences between ME and NM groups. Only cluster sizes >100 voxels are shown. Regions in red or blue color were larger or smaller, respectively, in the ME group.

**Figure 4:**
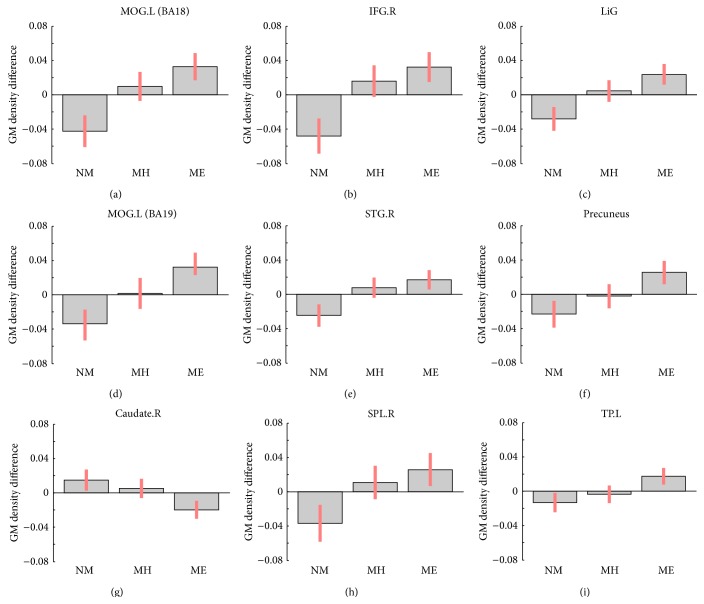
Gray matter density differences between nonmusic (NM), music hobby (MH), and music expert (ME) groups at the selected voxel (MNI coordinates are given in Table 2 for (a)–(g) and in the text for (h)-(i)). Vertical red bars indicate 90% confidence intervals. (a) Left middle occipital gyrus (BA 18); (b) right inferior frontal gyrus; (c) lingual gyrus; (d) left middle occipital gyrus (BA 19); (e) right superior temporal gyrus; (f) precuneus; (g) right caudate nucleus; (h) right superior parietal lobule; (i) left temporal pole.

GM, WM, and total brain volumes are summarized in Table 1. The *P* values obtained by ANOVA of all three groups are also provided. GM, WM, and total volume did not differ between groups. The surface map of brain regions with differing GM volumes is shown in Figure 1. Sagittal and horizontal cross sections are shown in Figures 2 and 3. Brain regions with different volumes between ME and NM groups are summarized in Table 2. The ME group had larger volumes in the bilateral LiG, left MOG (BA 18), and right IFG than in the NM group. The ME group also tended to have larger volumes in the left MOG (BA 19), right STG (BA 22), right anterior insula (AI), and precuneus. These regions showed larger clusters than the threshold size of 252. In contrast, the right caudate nucleus showed a trend to be smaller in the ME group than in the NM group. Comparison of relative GM volumes between the three groups is shown in Figure 4. This figure also included the right SPL (peak MNI: 30, −53, 59) (*k* = 242;* t* = 2.87) and left temporal pole (peak MNI: −44, 17, −42) (*k* = 203;* t* = 2.76), although below the cluster size threshold. In all regions ((a) through (i)), volumes for the MH group were in between the other two groups.

## 4. Discussion

In our study, the brain regions with the largest volumes in the ME group were higher-order sensory and association areas, which are not inherently music-proper areas. However, our finding is consistent with the fact that musical training requires higher-order cognitive and attentional functions [9, 11]. In the following section, we discuss the information processing required in musical training by associating the functions of the altered regions.

### 4.1. Inferior Frontal Gyrus

A study found that the left posterior IFG (including Broca's area) was larger in male symphony orchestra musicians [7]. The subjects (both musicians and controls) in this study showed a wide range of ages (26–66 years). Although the volume of Broca's area decreased with age, the decrement was much smaller or nullified in musicians (*P* = 0.44); therefore, the volume differed between groups at >45 years of age. In addition, VBM and deformation-based morphometry (DBM) studies detected significant differences in the right IFG between musicians and nonmusicians [5, 12]. Consistently with these studies, we observed a volumetric difference in the right posterior IFG. There could be several interpretations for this, as discussed below.

#### 4.1.1. Syntax Processing

The left posterior IFG is crucial for syntax processing in language and music [13, 14]. Although lateralization of the posterior IFG has been recognized for language [15], lateralization for music remains less clear. A cortical network comprising the IFG (BA 44), ventrolateral premotor cortex (PMC), and anterior STG has been implicated in the processing of musical structure [16], which is also a principal component of the language processing network [17]. Bilateral IFG activation was observed during a harmonic processing task in an fMRI study of nonmusicians with moderate musical training [18]. Furthermore, a magnetoencephalography study showed bilateral BA 44 activation for harmonically inappropriate chords [19]. Interestingly, a chord sequence paradigm activated BA 44 with right-hemispheric weighting [20]. Taken together, these previous studies suggest that music and language use a common network but with different lateralization tendencies, with language favoring the left hemisphere [13, 21]. Our analysis found a difference between the ME and NM groups in the right BA 44, the right-hemisphere homologue of Broca's area. A plausible interpretation of this is that the right posterior IFG is enlarged in the ME group due to the highly demanding syntax processing of musical training. However, this does not simply imply that left BA 44 processes lingual syntax, while right BA 44 processes musical syntax. There is the possibility that the left BA 44 did not differ because NM students were using language intensively. Consequently, only the right BA 44 would show a significant difference. Therefore, our result suggests that musical training has increased the right posterior IFG volume, potentially due to the involvement of this brain region in music syntax processing, and thereby supporting the previous suggestion.

#### 4.1.2. Cognitive Control

An fMRI study using a three-dimensional mental rotation task found that orchestral musicians had significantly increased activity in Broca's area, in addition to the visuospatial network, which was activated in both musicians and age-matched nonmusicians [22]. This finding suggests that Broca's area also contributes to the control of cognitive operations. The IFG (in particular the posterior part) is a node of the frontoparietal mirror neuron system that is suggested to play an important role in playing and listening to music through the observation of others' actions for understanding emotion and intention [23]. This brain region is also activated by the perception of faces with emotion [24]. More broadly, the IFG has been associated with cognitive control of memory [25], response selection/inhibition and other self-control functions [26], and cognitive flexibility [27]. Working memory is critical in music performance. Verbal and tonal working memory appear to share a common network comprising the IFG, PMC, and inferior parietal lobule, and musicians activate tonal working memory more strongly than nonmusicians [28]. Interestingly, musicians, but not nonmusicians, recruit other brain regions for tonal as well as verbal working memory. For tonal working memory, these regions include the left cuneus, right globus pallidus, right caudate nucleus, and left cerebellum [28]. Working memory and cognitive control in music connect many brain regions, which enables complex multitask performance. Therefore, hypothetically, enlargement of the posterior IFG in the ME group may reflect inherent involvement of cognitive control in musical training and performance.

#### 4.1.3. Creativity

Another interesting construct that may account for the difference in right BA 44 volume is “creativity.” Both the left and right IFG are correlated with verbal creativity [29]. Moreover, a functional connectivity study suggested that subjects with higher creativity have increased connectivity at rest between BA 44 in both hemispheres and the default mode network (DMN) [30]. They also showed that the right BA 44 has stronger connectivity with the left dorsolateral prefrontal cortex in subjects with higher divergent thinking ability. Therefore, the right BA 44 volume difference observed in our study may reflect, at least partially, a difference in creativity between the groups, although we did not assess creativity in our participants.

### 4.2. Visual and Visuospatial Areas

The ME group had larger volumes in bilateral LiG, left MOG (BA 18), and left MOG (BA 19) than in the NM group. This finding is novel to the best of our knowledge. These visual association areas have multiple functions that are considered relevant to music. Visual attention ability is enhanced in musicians compared with nonmusicians [31]. Harmonic processing activates different visual association areas for musicians and nonmusicians [32]. Bilateral LiG activation is associated with harmonic processing in musicians, but not naive subjects, suggesting that musical training changes visual representation of harmonies [33]. In a positron emission tomography study, LiG activation was associated with visual imagery of a subjects' hands playing an electronic piano with their eyes closed [34]. For playing musical instruments, dynamic spatial mapping from visual information is also critical. In this regard, the dorsal visual pathway (including the SPL) is likely important for music performance. Our present study shows that the SPL tends to be larger in the ME group. This is consistent with a previous study suggesting that the SPL mediates spatial mapping of reading music scores to fingers tapping a piano key [35]. This region was also activated in music perception, which requires selective attention [36]. Furthermore, melody processing and sight reading activate the SPL and intraparietal sulcus (IPS) [37, 38]. The bilateral IPS has been implicated as part of a multimodal network for systematic transformation of stimulus information [39]. Specifically, this study shows a positive correlation between bilateral IPS GM volume and relative pitch performance in healthy volunteers with a wide range of musical experiences.

### 4.3. Other Areas

The right STG (BA 22), or secondary auditory cortex, was larger in the ME group at a trend level. The right STG has been associated with pitch processing [5, 39]. Musical sophistication appears to cause a shift of musical perception from the right to the left hemisphere [40]. Musical memory bilaterally activates the STG [41]. STG activity is also modulated bilaterally by music with an affective tone, but with the right hemisphere weighted [42].

Right AI volume had a trend to be larger in the ME group than in the NM group. AI involvement in music has been consistently observed [34, 43]. Both singing and speech activate the AI, although singing activates the right AI and speech activates the left [44]. Processing of music-evoked emotions also activates the AI, with increased activation for emotionally mismatched displays compared with emotionally matched displays [45], suggesting that the AI plays an active role in monitoring the consistency of musical emotional information. Furthermore, this area is broadly associated with emotional awareness, interoception, body movement awareness, auditory and visual awareness of the moment, time perception, perceptual decision making, cognitive control, and performance monitoring [43]. All of these functions are relevant to music performance.

The ME group had larger precuneus volumes than the NM group, albeit at a trend level. Along with the posterior cingulate cortex, the precuneus is a core DMN node [46], and its function has been associated with visual imagery, episodic memory, and self-processing [47]. A recent study suggested that mental representation of an auditory scene involves the precuneus [33]. Moreover, scene construction consistently activates the precuneus [48]. Considered along with these findings, our result suggests that mental imagery or scene construction plays an important role in musical training.

We found that the right caudate nucleus was the only region smaller in the ME group than in the NM group. Interestingly, a recent VBM study reported that skilled ballet dancers have smaller motor areas [49]. The caudate nucleus constitutes the cortico-striato-pallido-thalamo-cortical loop for sensorimotor control [50]. There is the possibility that ballet training shapes the loop circuit so that efficient signal transmission through the circuit is enabled with a smaller volume. Another study suggested that perirolandic areas and striatal volumes are reduced by musical training, while several cortical areas such as the IFG, Heschl's gyrus, IPL, and cerebellum show increased volumes in expert musicians [9]. The reduced areas are sensorimotor areas and may follow the principle of parsimony [9]. Although further study is needed, our result supports this notion.

### 4.4. Limitations

A major limitation of our study is the sample size. Our analysis found group differences at the level of *P* < 0.001, uncorrected. Increasing the threshold by correcting for multiple comparisons found no differences. Therefore, we employed this threshold level conventionally, according to several previous studies [8, 9, 39, 51]. We divided the nonmusic students into two groups because we could then determine if the volumetric differences were graded depending on musical training intensity. The degree of the differences will be ascertained using a larger sample size.

## 5. Conclusions

Using VBM, we observed larger GM volumes in the right IFG, left MOG (BA 18), and bilateral LiG in the ME group than in the NM group. Right IFG enlargement may be associated with musical syntax processing or, more generally, cognitive control in musical training and performance. Visual area enlargement in the ME group may be associated with multiple functions relevant to music including visual attention, harmonic processing, and visual imagery. These volumetric differences are considered to be caused by neuroplasticity during long and continuous musical training periods because the MH group showed intermediate volumes in these regions.

## Figures and Tables

**Table 1:** Gray matter (GM), white matter (WM), and total brain volumes (all in mL) and *P* values obtained by ANOVA between all three groups. NM: nonmusic; MH: music hobby; ME: music expert.

	NM		MH		ME		*P* value
	Mean	SD	Mean	SD	Mean	SD	
GM	676.6	46.3	697.9	65.8	685.6	48.1	0.533
WM	441.5	54.8	444.5	41.1	465.8	47.0	0.225

Total	1118.1	82.2	1142.4	83.9	1151.4	79.9	0.469

**Table 2:** Brain regions and peak locations (in MNI coordinates) that differed between music expert and nonmusic groups. Brain regions with cluster sizes, *k* > 252 voxels (expected voxels per cluster), are shown. ^∗^ *P* < 0.001 (uncorrected) for two-sample *t*-tests; otherwise, *P* < 0.01 (uncorrected).

Brain region	*x*	*y*	*z*	Cluster size (in voxels)	*t* value
Music experts > Nonmusic					
Left MOG (BA18)	−27	−86	−2	383	4.10^∗^
Right IFG (BA44)	44	11	23	566	4.00^∗^
Bilateral LiG	−2	−77	−6	1389	3.74^∗^
Left MOG (BA19)	−33	−89	17	313	3.34
Right STG (BA22)	53	11	−2		3.25
Right AI	38	24	12	734	3.14
Right AI/IFG	41	17	6		3.00
Precuneus	0	−48	53	373	3.14

Nonmusic > Music experts					
Right caudate nucleus	18	14	9	253	2.87
